# Treatment strategy of adding transcatheter arterial chemoembolization to sorafenib for advanced stage hepatocellular carcinoma

**DOI:** 10.1002/cnr2.1294

**Published:** 2020-10-13

**Authors:** Wei‐Chen Lee, Hao‐Chien Hung, Jin‐Chiao Lee, Yu‐Chao Wang, Chih‐Hsien Cheng, Tsung‐Han Wu, Chen‐Fang Lee, Ting‐Jung Wu, Hong‐Shiue Chou, Kun‐Ming Chan

**Affiliations:** ^1^ Division of Liver and Transplantation Surgery, Department of General Surgery Chang‐Gung Memorial Hospital Linkou Taiwan; ^2^ Department of Medicine Chang‐Gung University College of Medicine Taoyuan Taiwan

**Keywords:** hepatocellular carcinoma, neutrophil‐to‐lymphocyte ratio, sorafenib, transcatheter arterial chemoembolization

## Abstract

**Background:**

Therapeutic effect and immunosuppressor cell alteration in adding transcatheter arterial chemoembolization (TACE) to sorafenib for advanced stage hepatocellular carcinoma (HCC) remain unclear.

**Aims:**

To examine the therapeutic effect and immunosuppressor cell alteration in adding TACE to sorafenib.

**Methods:**

Forty‐four advanced stage HCC patients were divided into group A (n = 17) treated by sorafenib (400‐600 mg/day) alone and group B patients (n = 27) treated by sorafenib and TACE. The frequency of regulatory T‐cells and myeloid‐derived suppressor cells (MDSC), and patients' outcomes were examined. Advanced HCC patients' survival was improved by adding TACE to sorafenib if N/L was reduced from ≥2.5 to <2.5 by TACE.

**Results:**

The median (interquartile) follow‐up for all patients was 8.5 (3.5 to 15.5) with a range from 1 to 71 months. The median (interquartile) survival was 5.0 (2.3‐11.3) months for group A and 11.0 (5.0‐19.0) months for group B patients (*P* = .024). In group A, the patients (n = 8) with neutrophil‐to‐lymphocytes ratio (N/L) < 2.5 had better survival than the patients (n = 9) with N/L ≥ 2.5 (*P* = .006). In group B, 6 of 13 patients with N/L ≥ 2.5 had N/L reduction to <2.5 after combination therapy of sorafenib and TACE, and their 6‐month, 1‐year and 2‐year survival were improved (*P* = .013). For immune cell examination, the frequency of CD4^+^ and CD8^+^ T‐lymphocytes, regulatory T‐cell and MDSC were not altered by sorafenib treatment. However, actual number of lymphocytes had a tendency to increase (from 978.5 ± 319.4/mm^3^ prior to treatment to 1378.0 ± 403.3/mm^3^, *P* = .086) for the patients with N/L reduction.

**Conclusion:**

Immunosuppressor cells were not altered by sorafeinb. Patients' survival was improved if N/L ≥ 2.5 was reduced to <2.5 by TACE.

**Abbreviations**
BCLC: Barcelona Clinic Liver Cancer
HCC: hepatocellular carcinoma
MDSC: myeloid‐derived suppressor cell
N/L: neutrophil‐to‐lymphocytes ratio
TACE: transcatheter arterial chemoembolization
Treg: regulatory T‐cell

## 1 INTRODUCTION

Hepatocellular carcinoma (HCC) is the most common primary malignant tumor in the liver. HCC in early stage is silent and difficult to be found unless the patients with chronic liver diseases are regularly screened for liver tumors. Therefore, HCC may be already in advanced stage once upon the tumors are found. Even HCC is found in early stage and treated by liver resection or radiofrequency ablation, HCC is easy to recur and progress into advanced stage.^1^, ^2^ Therefore, HCC is the fifth most common malignancy in the world, but is the third common cause of cancer death.^3^, ^4^ How to treat advanced stage HCC and prolong patients' survival is more crucial than the treatments for early stage HCC.

When HCC is advanced with portal vein thrombus or extrahepatic spread and patients' Eastern Cooperative Oncology Group (ECOG) performance status is 1‐2, systemic treatment is recommended in accordance with Barcelona Clinic Liver Cancer (BCLC) staging and treatment strategy.^5^ In Hong Kong cancer staging, HCC with portal vein thrombus or extrahepatic spread is stage IVa and the therapy is the same as BCLC staging.^6^ Sorafenib is the first molecular targeting drug approved to treat advanced stage HCC. In the Sharp and the Asia‐Pacific randomized clinical trials, sorafenib was applied to treat advanced stage HCC and overall survival could be prolonged for 2.7‐2.8 months.^7^, ^8^ However, the median overall survival time in these two studies was only 6.5‐10.7 months. How to further prolong survival of advanced stage HCC patients is challenging.

Multimodality therapy may be one of the options for advanced stage HCC treatment. Based on intension‐to‐treat, advanced stage HCC patients may receive sorafenib or immunotherapy for systemic treatment and transcatheter arterial chemoembolization (TACE) for intra‐liver tumor local treatment.^9^, ^10^, ^11^ Radiotherapy may also be added for intravascular tumor‐thrombus treatment. Whether local therapy such as TACE yields clinical benefit for advanced stage HCC remains to be determined.

Immunity is known to be important for cancer treatment. Alternation of immune system in the patients with large HCC tumor burden is already known. CD4^+^CD25^+^Foxp3^+^ regulatory T (Treg) cells are immunosuppressive cells and stay in cancer ^12^, ^13^. HLA‐DR^−^CD33^+^ myeloid‐derived suppressor cells (MDSC) is another immunosuppressive cell of myeloid origin and characterized by production of reactive oxygen, nitrogen species and arginase I to suppress immunity.^14^, ^15^, ^16^ In this study, we would examine the alteration of immune cells and clinical outcomes while advanced stage HCC was treated by sorafenib with or without TACE.

## 2 PATIENTS AND METHODS

### 2.1 Patients and treatments

Forty four patients who had advanced stage HCC with main portal vein thrombus, hepatic vein thrombus, inferior vena cava (IVC) thrombus or extrahepatic metastasis were included. All the patients received sorefenib (400‐600 mg/day) for systemic treatment. TACE was applied to treat the patients whose tumor burden in the liver remained large. Radiotherapy was applied to treat the patients whose tumors formed thrombus in main portal vein, hepatic vein or IVC. Blood samples from these advanced HCC patients were taken for immune cell analysis prior to sorafenib treatment and 1 month after sorafenib administration. All the patients signed informed consent of providing blood samples for immune cell analysis and clinical research. Clinical characteristics of these patients were recorded, including age, gender, liver function and white blood cell count. The patients were divided into two groups: group A, the patients treated by sorafenib without TACE; group B, the patients treated by sorafenib with TACE. This study was confirmed to the ethical guidelines of the 1975 Declaration of Helsinki and was approved by institutional review board of Chang‐Gung Memorial Hospital (IRB No. 101‐3552B).

### 2.2 Phenotypic analysis of immune cells

Ten milliliters of peripheral blood was drawn prior to sorafenib treatment and 1 month after sorafenib administration. Peripheral blood monocytes (PBMC) were isolated by Ficoll‐Hypaque (GE Healthcare, Uppsala, Sweden) density centrifugation. The phenotypic analysis of immune cells was performed by flow cytometry after the cells were stained with fluorescence‐conjugated monoclonal antibodies. The surface monoclonal antibodies included anti‐CD4 (RPA‐T4 clone; PharMingen, San Diego, California) anti‐CD8 (RPA‐T8 clone; PharMingen), anti‐CD33 (WM 53 clone, PharMingen), anti‐HLA‐DR (TU36 clone, PharMingen), anti‐CD40 (5C3 clone, PharMingen) and anti‐CD86 (FUN‐1 clone, PharMingen). The intracellular Foxp3 was stained by fluorescence‐conjugated rat anti‐human foxp3 (eBioscience, San Diego, California). The expression of these molecules was analyzed by cytofluorography employing a Beckman Coulter NAVIOS flow cytometer (Beckman Coulter Co., Indianapolis, Indiana). Regulatory T‐cells were identified as positive for CD4 and foxp3. MDSC was identified as positive for CD33 and negative for HLA‐DR.

### 2.3 Intracellular cytokine staining

Brefeldin A (5 μg/mL) was added into the in vitro culture of PBMC for 4 hours. The cell was fixed by 2% paraformaldehyde and permeabilized by saponin (0.5%). Intracellular cytokine was analyzed by cytofluorography employing a Beckman Coulter NAVIOS flow cytometer (Beckman Coulter Co.) after the intracellular cytokine was stained by PE‐conjugated mouse anti‐human IFN‐γ (1/50×; PharMingen).

### 2.4 TACE

Under adequate local anesthesia, an angiographic sheath was inserted into right femoral artery as a working channel for angiographic catheter. The angiographic catheter was threaded into celiac trunk and common hepatic artery to perform angiographic study of liver tumors. Then, the tumor‐located segmental arteries were cannulated super‐selectively with 3 French coaxial micro‐catheter and embolized by emulsion of lipiodol mixed with 10‐20 mg adriamycin (20 mg epirubicin) or elution beads loading with adriamycin. TACE was performed every 2‐3 months unless the liver function was deteriorated to Child‐Pugh score >8 or the tumors were already well‐embolized.

### 2.5 Survival time

The patients were followed up at outpatient clinic every month after sorafenib was prescribed. Dynamic computed tomography (CT) of the liver was performed every 3 months to see the tumor status. Overall survival was measured from the date of sorafenib prescribed to date of last following up or patients' death.

### 2.6 Biostatistics analysis

Comparisons of categorical variables were determined by the *χ* ^2^ tests. Significance of differences between groups was determined by an unpaired or paired Student's *t* test. Overall survival rates after prescription of sorafenib were calculated using the Kaplan‐Meier method and comparison between groups was performed through log‐rank test. The statistical analyses were all performed with SigmaPlot 12.3 software for Windows (Systat Software, Inc., San Jose, California). *P* value below .05 was considered to be significantly different.

## 3 RESULTS

### 3.1 Patients

Forty four patients who received sorafenib (400‐600 mg/day) treatment were included in this study. These 44 patients were divided into group A patients (n = 17) who had sorafenib systemic treatment only, and group B patients (n = 27) who had sorafenib systemic treatment combined with TACE. In group A, 15 (88.2%) patients were male and 16 (94.1%) patients' liver function was in Child A classification. In group B, 25 (92.6%) patients were male and 26 (96.3%) patients' liver function was in Child A classification. Patients' age between group A and B was not different. Most of the patients in group A and B were infected with hepatitis B or C (Table 1). Six patients in group A and 16 patients in group B had additional radiotherapy for right portal vein, left portal vein or IVC thrombus with a dosage ranging from 2000 to 5100 centigray.

**TABLE 1:** The characteristics of the patients received sorafenib with or without TACE

	Sorafenib	Sorafenib with TACE	*P*
Number of patients	17	27	
Gender (M/F)	15/2	23/4	1.000
Age (IQ)(range)	60 (53‐65.5) (35‐760)	59 (51‐62) (38‐78)	.649
Child classification			1.000
A	16	26	
B	1	1	
hepatitis			.359
B	13	19	
C	3	2	
B + C	1	3	
N	0	3	
Radiotherapy (yes/no)	6/11	16/11	.216
Tumor status			
Number	2.7 ± 2.0	3.4 ± 2.2	.489
Diameter (cm)	6.5(3.4‐12.5) (2, 3, 4, 5, 6, 7, 8, 9, 10, 11, 12, 13, 14, 15)	6.2(3.5‐13.5)(1.8‐14.2)	1.000
Lung metastasis	9 (52.9%)	12 (44.4%)	.811
PVT	5 (29.4%)	11 (40.7%)	.611
LN metastasis	1 (11.8%)	2 (7.4%)	.634
HVT + IVCT	3 (17.6%)	2 (7.4%)	.359
Carcinomatasis	1 (5.9%)	2 (7.4%)	1.000
Neutrophil (%) [median(IQ)(range)]			
Before treatment	63.9(52‐69.6)(44.3‐80.7)	65.8(57.6‐73.4)(42.6‐84.9)	.596
Post‐treatment^a^	67.7(54.2‐72.2)(41.4‐90.7)	64.7(57.8‐73.5)(45.2‐93.0)	.789
Lymphocytes (%)			
Before treatment	24.3(21.5‐36.3)(11.5‐47.3)	20.3(15.9‐34.2)(7.3‐46.8)	.394
Post‐treatment^a^	19.5(16.0‐32.6)(4.6‐45.2)	23.0(13.4‐33.2)(4.3‐42.2)	.803
N/L			
Before treatment	2.72(1.43‐3.19)(0.94‐7.02)	3.29(1.66‐4.53)(0.92‐11.63)	.394
Post‐treatment^a^	3.50(1.58‐3.86)(0.92‐19.72)	3.00(1.51‐5.63)(0.21‐21.6)	.978

Abbreviations: HVT, hepatic vein thrombus; IQ, interquartile; IVCT, inferior vena cava thrombus; LN, lymph node; N, negative for hepatitis B and C; N/L, neutrophil‐to‐lymphocyte ration; PVT, portal vein thrombus; TACE, transcatheter arterial chemoembolization.

^a^ One month after treatment.

### 3.2 Survival rates for group A and B patients

**FIGURE 1:**
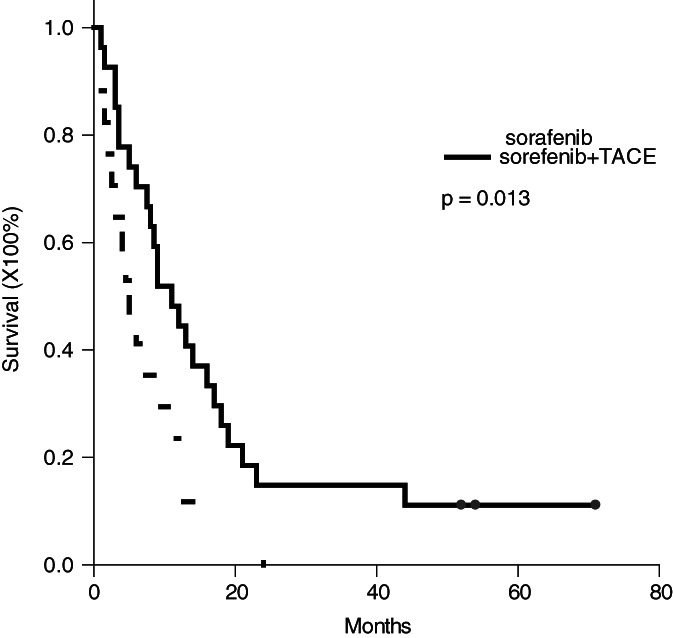
Kaplan–Meier survival curve of the patients treated by sorafenib with or without TACE. The 3‐month, 6‐month, 1‐year and 2‐year survival rates were 64.7%, 41.2%, 11.8% and 0% for the patients treated by sorafenib without TACE and 85.2%, 70.4%, 44.4% and 14.8% for the patients treated by sorafenib with TACE, respectively (*P* = .013)

The median (interquartile) follow‐up for all patients was 8.5 (3.5 to 15.5) months with a range from 1 to 71 months. The median (interquartile) survival was 5.0 (2.3‐11.3) months for group A patients, and 11.0 (5.0‐19.0) months for group B patients (*P* = .024). When the survival was calculated using the Kaplan‐Meier method, the 3‐month, 6‐month, 1‐year and 2‐year survival rates were 64.7%, 41.2%, 11.8% and 0%, respectively, for group A patients, and 85.2%, 70.4%, 44.4% and 14.8%, respectively, for group B patients (*P* = .013, Figure 1). This result showed that the patients treated by a combination of sorafenib and TACE had better survival rate than the patients treated by sorafenib alone.

### 3.3 Survival rates in accordance with neutrophil to lymphocyte ratio

**FIGURE 2:**
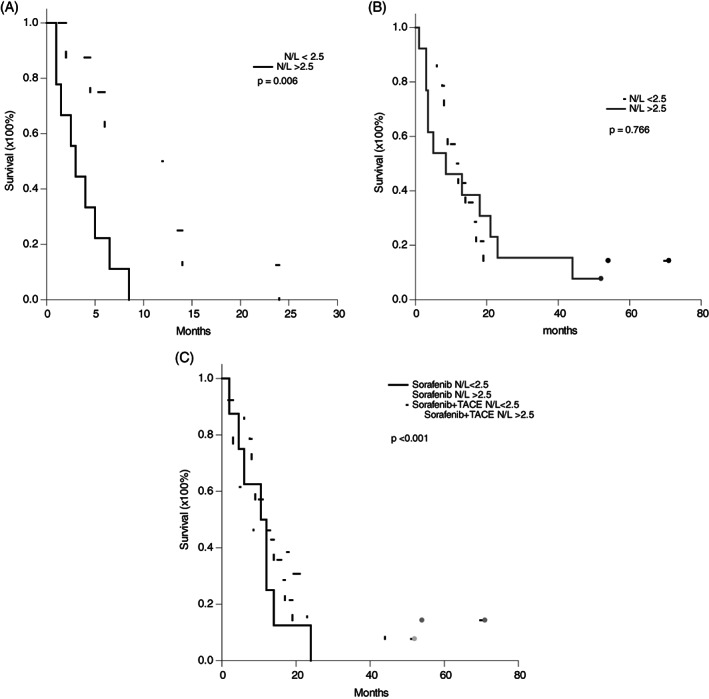
Kaplan–Meier survival curve of the patients based on neutrophil‐to‐lymphocyte ratio. A, For the patients in group A treated by sorafenib, the 3‐month, 6‐month, 1‐year and 2‐year survival for the patients with N/L < 2.5 were 87.3%, 62.5%, 25.0% and 0%, respectively, compared to 44.4%, 22.2%, 0% and 0% for the patients with N/L ≥ 2.5 (*P* = .006). B, For the patients in group B treated by sorafenib with TACE, the 3‐month, 6‐month, 1‐year and 2‐year survival for the patients with N/L < 2.5 were 92.9%, 85.7%, 42.9% and 14.3%, respectively, compared to 76.9%, 53.8%, 46.2% and 15.4% for the patients with N/L ≥ 2.5 (*P* = .766). C, Taking together, the survival rate was worst in the group A patients with N/L ≥ 2.5 (*P* < .001). The survival rates were not different among the patients in group A with N/L < 2.5, in group B with N/L < 2.5, and in group B with N/L ≥ 2.5 (*P* = .860)

In this study, 2.5 of N/L was applied as the cutoff point to further divide group A and B patients into subgroups.^17^ In group A patients, 8 patients had N/L < 2.5 and 9 patients had N/L ≥ 2.5 prior to sorafenib treatment. After 1 month of sorafenib treatment, N/L was not altered in these patients. The patients with N/L < 2.5 prior to treatment had much better prognosis than the patients with N/L ≥ 2.5. The 3‐month, 6‐month, 1‐year and 2‐year survival for the patients with N/L < 2.5 were 87.3%, 62.5%, 25.0% and 0%, respectively, compared to 44.4%, 22.2%, 0% and 0% for the patients with N/L ≥ 2.5 (*P* = .006, Figure 2A). In group B patients, 14 patients had N/L < 2.5 and 13 patients had N/L ≥ 2.5 prior to sorafenib treatment. The 3‐month, 6‐month, 1‐year and 2‐year survival for the 14 patients with N/L < 2.5 were 92.9%, 85.7%, 42.9% and 14.3%, respectively, compared to 76.9%, 53.8%, 46.2% and 15.4% for the 13 patients with N/L ≥ 2.5 (*P* = .766, Figure 2B). This result showed that the survival rates were not different between the patients with N/L < 2.5 or N/L ≥ 2.5 in group B patients. When the survival rates of the patients with N/L < 2.5 in group A and B were compared, the survival rates were not different between group A and B patients (Figure 2C, *P* = .765). But, when the patients with N/L ≥ 2.5 in group A and B were compared, the 3‐month, 6‐month, 1‐year and 2‐year survival in group A patients with N/L ≥ 2.5 were worse (Figure 2C, *P* < .001).

### 3.4 Clinical significance of neutrophil to lymphocyte ratio reduction

**FIGURE 3:**
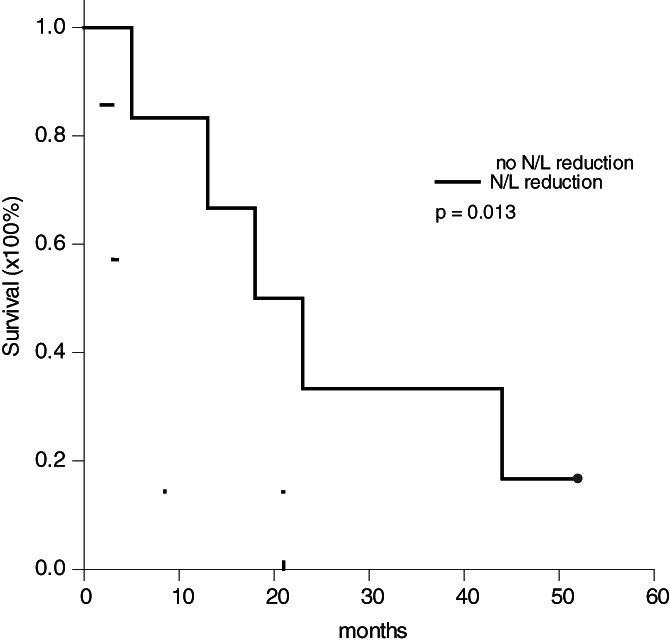
Kaplan‐Meier survival curve of the patients with neutrophil‐to‐lymphocyte ratio reduction. Among the 13 patients N/L ≥ 2.5 in group B, 6 (46.2%) patients had N/L reduction after combination therapy of sorafenib and TACE. The 3‐month, 6‐month, 1‐year and 2‐year survival for these 6 patients were 83.3%, 83.3%, 66.7% and 33.3%, respectively, compared to 57.1%, 28.6%, 14.3% and 0%, respectively, for the other 7 patients (*P* = .013)

To determine why the patients with N/L ≥ 2.5 in group B had a better survival than the patients with N/L ≥ 2.5 in group A, the alternation of N/L after treatment was investigated. There were no patients having N/L reduction after one‐month administration of sorafenib in group A. In the other hand, there were 6 of 13 patients having N/L reduction to below 2.5 after combination therapy of sorafenib and TACE in group B. The 3‐month, 6‐month, 1‐year and 2‐year survival for these 6 patients in group B were 83.3%, 83.3%, 66.7% and 33.3%, respectively, which were much better than 57.1%, 28.6%, 14.3% and 0%, respectively, for the other 7 patients without N/L reduction (*P* = .013, Figure 3). Therefore, the patients with N/L ≥ 2.5 in group B having better survival than in group A was due to 46.2% of group B patients having N/L reduction after sorafenib combined with TACE treatment.

### 3.5 Effects of sorafenib with or without TACE on immune cells

To determine whether sorafenib had effects on immune cells, PBMC from group A and B patients prior to treatment and after treatment was analyzed by flow cytometry. The results showed that the frequency of CD4^+^, CD8^+^, CD4^+^IFN^+^ and CD8^+^IFN^+^ T‐lymphocytes were not different between prior to sorafenib treatment and after sorafenib treatment. The frequency of immunosuppression cells, regulatory T‐cell and MDSC, were not different prior to and after treatment, either. The expression of costimulatory molecules on antigen‐presenting cells were not changed. (Table 2) Taking together, sorafenib with or without TACE did not change the frequency of immune cells. Further, we examined the actual number of lymphocytes prior to treatment and after treatment for group B patients with N/L ≥ 2.5. These patients were divided into post‐treatment N/L reduction and non‐reduction groups. The results showed that the actual number of lymphocytes were not different between reduction and non‐reduction groups prior to treatment (978.5 ± 319.4 vs 885.5 ± 492.0/mm^3^, *P* = .703). After treatment, the actual number of lymphocytes had a tendency to increase in N/L reduction group (from 978.5 ± 319.4/mm^3^ to 1378.0 ± 403.3/mm^3^, *P* = .086), and to decrease in non‐reduction groups (from 885.5 ± 492.0/mm^3^ to 568.5 ± 220.1/mm^3^, *P* = .059). Therefore, the patients with N/L reduction after treatment had higher actual number of lymphocytes than the patients without N/L reduction (1378.0 ± 403.3/mm^3^ vs 885.5 ± 492.0/mm^3^, *P* = .002).

**TABLE 2:** The alteration of immune cells after sorafenib with or without TACE treatment

	Sorafenib [mean ± SD or median(IQ)]	*P*	Sorafenib + TACE [mean ± SD or median(IQ)]	*P*
CD4^+^ T‐cell (%)		.714		.744
Pre‐treatment	39.2 ± 12.8		34.1 ± 12.6	
Post‐treatment	38.4 ± 11.3		34.6 ± 11.8	
CD8^+^ T‐cell (%)		.708		.362
Pre‐treatment	24.6 ± 8.3		24.4 ± 9.4	
Post‐treatment	25.3 ± 7.7		27.1 ± 12.3	
CD86 (%)		.736		.122
Pre‐treatment	20.73 ± 15.89		22.36 ± 16.32	
Post‐treatment	23.81 ± 12.28		17.91 ± 12.05	
Regulatory T‐cell (%)		.097		.820
Pre‐treatment	3.24 ± 2.58		3.84 ± 7.45	
Post‐treatment	6.02 ± 5.57		3.09 ± 2.97	
MDSC (%)		.097		.773
Pre‐treatment	3.18 (1.52‐7.44)		3.90 (1.80‐7.31)	
Post‐treatment	4.15 (2.36‐8.40)		3.72 (1.83‐7.60)	
CD4^+^IFN‐**γ** ^+^ (%)		1.000		.077
Pre‐treatment	4.08 (1.87‐6.09)		2.41 (1.06‐5.33)	
Post‐treatment	3.54 (0.08‐9.08)		1.68 (0.21‐3.74)	
CD8^+^IFN‐**γ** ^+^ (%)				.257
Pre‐treatment	10.87 ± 13.15	.361	5.63 ± 7.05	
Post‐treatment	9.85 ± 6.68		5.54 ± 4.64	

Abbreviations: IFN, Interferon; IQ, interquartile; MDSC, myeloid‐derived suppressor cell; SD, standard deviation; TACE, transcatheter arterial chemoembolization.

## 4 DISSCUSSION

Treatment for advanced stage HCC is difficult and challenging. Systemic treatment is recommended in accordance with BCLC staging and therapeutic strategy for advanced stage HCC. Sorafenib is the first systemic molecular target therapy recommended to treat advanced stage HCC patients because sorafenib could prolong patients' survival for 2.3‐2.8 months in phase III ramdomized trials.^7^, ^8^ TACE is not recommended at this stage because locoregional treatment is not recognized as an effective treatment for advanced stage HCC which is recognized as a systemic disease rather than local tumors. However, TACE is applied as the first‐line treatment in nearly 50% of advanced stage HCC patients in real world clinical practice.^18^ Furthermore, locoregional treatment of tumors to decrease tumor burden may alleviate the tumor effects on hosts' immunity and maintain anti‐tumor immunity. This may have clinical benefits for the patients. In a meta‐analysis study, TACE improved 6‐month and 1‐year survival for advanced stage HCC patients who had portal vein thrombus.^19^ Therefore, we remain selectively to perform TACE for advanced stage HCC patients who still had large tumor‐burden in the liver.

Sorafenib combined with TACE was a more effective treatment than sorafenib alone. In this study, the patients receiving the combination treatment of sorafenib and TACE had a better survival rate than the patients treated with sorafenib alone. The median survival was prolonged from 5 months to 11 months and 14.8% of the patients survived for more than 2 years. In a phase III randomized STAH study conducted by Dr Part, et al., advanced stage HCC was treated by sorafenib with or without TACE.^20^ During study period, there were no difference in median survival for the patients treated by sorafenib alone or combined with TACE although the patients treated by combination of sorafenib and TACE had better time‐to‐progression survival. However, in post hoc follow‐up, the patients treated by combination of sorafenib and TACE had a better median overall survival than the patients treated with sorafenib alone. This implied that TACE brought survival benefits to advanced stage HCC patients even distant metastasis or portal vein thrombus existed.

**FIGURE 4:**
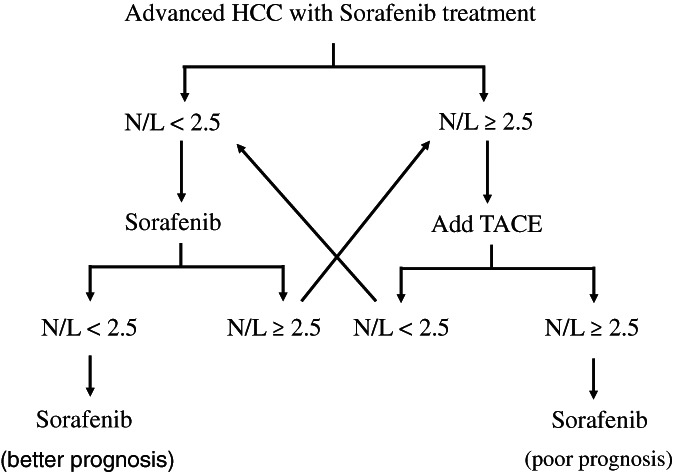
The treatment strategy scheme for advanced stage HCC

Nevertheless, TACE is an invasive procedure and patients may be suffered from the adverse effects of TACE. Because advanced stage HCC patients are already sick, it is better to choose patients who may get benefits from TACE to perform TACE. In accordance with the results of this study, there were no survival difference between group A and group B patients whose N/L was <2.5; that is, TACE in group B patients with N/L <2.5 did not showed clinical benefits. For the patients with N/L ≥2.5 prior to treatment, there was no patients in group A having N/L reduction after a‐month of sorafenib treatment; in the other hand, almost half of the patients in group B had N/L reduction and their survival was improved. Undoubtedly, TACE can be applied to advanced stage HCC patients whose N/L was ≥2.5, and the survival will be improved if N/L can be reduced. The suggested treatment strategy is showed in Figure 4.

Neutrophil to lymphocyte ratio (N/L) could be applied as a predictor of prognosis and a guidance of performing TACE for advanced stage HCC patients. When 2.5 of N/L was employed as the cutoff point for HCC patients,^17^ the patients in group A with N/L below 2.5 had significantly better survival rate than the patients with N/L ≥ more than 2.5. In group B patients, 6 (46.2%) patients with high levels of N/L had N/L reduction after combination therapy of sorafenib and TACE, and their survival was improved. N/L reflects the systemic inflammation status. Chronic inflammation is a risk factor of cancers and also a significant component of tumor progresssion.^21^, ^22^ The development of HCC are frequently related to hepatitis B, hepatitis C or alcohol‐associated inflammation or cirrhosis. As anti‐cancer immunity is mainly mediated by T‐lymphocytes, N/L may be used as a surrogate of hosts' immunity. In the literature, N/L was reported as a predictor of prognosis of hepatectomy for HCC, liver transplantation for HCC or even other treatments for HCC.^23^, ^24^, ^25^ This study confirmed that reduction of N/L from high levels could be employed as a well sign to indicate the treatment efficacy of sorafenib combined with TACE.

Anti‐cancer immunity is a complicated network of immune cells to combate tumors. In this study, T‐lymphocytes, immunosuppressor cells and the expression of costimulatory molecules on antigen‐presenting cells were analyzed. The results clearly showed the sorafenib did not change the percentages of CD4^+^ T‐cells, CD8^+^ T‐cells, regulatory T‐cells and myeloid‐derived suppressor cells in peripheral blood. When TACE was applied to treat the patients, the percentages of CD4^+^ T‐cells, CD8^+^ T‐cells, regulatory T‐cells and myeloid‐derived suppressor cells were not significantly changed, either. However, for the patients with reduction of high N/L, the total number of lymphocytes was increased and the prognosis was improved. This implied that total population of T‐lymphocytes was very important to control the diseases.

The limitation of this study was the small number of the patients and wide variety of the patients. However, it is a real world data and clearly shows sorafenib combined with TACE is better than sorafenib alone, particularly for the patients with N/L ≥ 2.5.

In conclusion, cutoff value 2.5 of N/L can be used as a surrogate of prognosis for advanced stage HCC. Adding TACE to sorafenib treatment for advanced stage HCC patients yields an improvement of patients' survival when the patients had N/L reduction from N/L ≥ 2.5. Although sorefenib and TACE did not change the distribution of immune cells in the patients with advanced stage HCC, decrease of tumor burden by TACE may have the chance to reduce N/L, increase the number of T‐lymphocytes, and improve prognosis.

## AUTHOR CONTRIBUTIONS

**Wei‐Chen Lee:** Conceptualization; writing‐original draft; writing‐review and editing. **Hao‐Chien Hung:** Formal analysis; investigation. **Jin‐Chiao Lee:** Data curation; investigation. **Yu‐Chao Wang:** Data curation; investigation. **Chih‐Hsien Cheng:** Investigation; methodology. **Tsung‐Han Wu:** Formal analysis; investigation. **Chen‐Fang Lee:** Investigation; methodology. **Ting‐Jung Wu:** Formal analysis; investigation. **Hong‐Shiue Chou:** Formal analysis; investigation. **Kun‐Ming Chan:** Formal analysis; investigation; methodology.

## CONFLICT OF INTEREST

There is no conflict of interest among authors.

## ETHICS STATEMENT

All the patients signed informed consent of providing blood samples for immune cell analysis and clinical research. This study was confirmed to the ethical guidelines of the 1975 Declaration of Helsinki and was approved by institutional review board of Chang‐Gung Memorial Hospital (IRB No. 101‐3552B).

## Data Availability

The data that support the findings of this study are available from the corresponding author, W.‐C. Lee, upon reasonable request.
